# A novel extraction-free dual HiFi-LAMP assay for detection of methicillin-sensitive and methicillin-resistant *Staphylococcus aureus*

**DOI:** 10.1128/spectrum.04133-23

**Published:** 2024-02-20

**Authors:** Xiuli Zhao, Yi Zeng, Beibei Yan, Yanping Liu, Yueqin Qian, Aiping Zhu, Yongjuan Zhao, Xiaoling Zhang, Chiyu Zhang, Zhenzhou Wan

**Affiliations:** 1 Medical Laboratory of Taizhou Fourth People’s Hospital, Taizhou, China; 2 Shanghai Public Health Clinical Center, Fudan University, Shanghai, China; University of Mississippi Medical Center, Jackson, Mississippi, USA

**Keywords:** *S. aureus*, MRSA, HiFi-LAMP, POCT, specificity, *mecA *gene, *nuc *gene

## Abstract

*Staphylococcus aureus* (*S. aureus*) is a leading cause of bacteremia and blood stream infections. Methicillin-resistant *S. aureus* (MRSA) that first appeared in 1961 often caused hospital-acquired infections (HAIs) and community-acquired infections (CAIs) and was associated with high mortality rate. Accurate and rapid point-of-care testing (POCT) of MRSA is crucial for clinical management and treatment of MRSA infections, as well as the prevention and control of HAIs and CAIs. Here, we reported a novel extraction-free dual HiFi-LAMP assay for discriminative detection of methicillin-susceptible *S. aureus* and MRSA. The dual HiFi-LAMP assay can detect 30 copies/reaction of *nuc* and *mecA* genes with detection limits of 147 and 158 copies per 25 µL reaction, respectively. A retrospective clinical evaluation with 107 clinical *S. aureus* isolates showed both sensitivity and specificity of 100%. A prospective clinical evaluation with 35 clinical samples revealed a specificity of 100% and a sensitivity of 92.3%. The dual HiFi-LAMP assay can detect almost all *S. aureus* samples (141/142; 99.3%) within 20 min, implying that the entire HiFi-LAMP assay (including sample process) can be completed within 40 min, extremely significantly shorter than 3–5 days by the traditional clinical microbial culture and antibiotic susceptibility testing. The novel extraction-free dual HiFi-LAMP assay can be used as a robust POCT tool to promote precise diagnosis and treatment of MRSA infections in hospitals and to facilitate surveillance of MRSA at hospital and community settings.

**IMPORTANCE:**

Methicillin-resistant *Staphylococcus aureus* (MRSA) was associated with high mortality rate and listed as a “priority pathogen” by the World Health Organization. Accurate and rapid point-of-care testing (POCT) of MRSA is critically required for clinical management and treatment of MRSA infections. Some previous LAMP-based POCT assays for MRSA might be questionable due to their low specificity and the lack of appropriate evaluation directly using clinical samples. Furthermore, they are relatively tedious and time-consuming because they require DNA extraction and lack multiplex detection capacity. Here, we reported a novel extraction-free dual HiFi-LAMP assay for discriminative detection of MRSA and methicillin-susceptible *S. aureus*. The assay has high specificity and sensitivity and can be completed within 40 min. Clinical evaluation with real clinical samples and clinical isolates showed excellent performance with 100% specificity and 92.3%–100% sensitivity. The novel extraction-free assay may be a robust POCT tool to promote precise diagnosis of MRSA infections and facilitate surveillance of MRSA at hospital and community settings.

## INTRODUCTION

As a common highly virulent clinical bacterium, *Staphylococcus aureus* (*S. aureus*) is a leading cause of bacteremia and blood stream infections, including skin and soft tissue infections, suppurative arthritis, otitis media, osteomyelitis, periprosthetic joint infections, and so on (1–3). The mortality rate in *S. aureus* bacteremia is reported to be 10–30% (4). Methicillin was first used for the treatment of *S. aureus* infection in 1959; however, the methicillin-resistant *S. aureus* (MRSA) quickly emerged in the United Kingdom within the subsequent 2 years (5). MRSA often causes hospital-acquired infections (HAIs) and community-acquired infections (CAIs) and is associated with a higher in-hospital mortality rate than methicillin-susceptible *S. aureus* (MSSA) (2, 3). Therefore, MRSA has become a major public health threat globally (6, 7).

Rapid and accurate detection of MSSA and MRSA is critical for patient management and antimicrobial treatment (8, 9). Blood culture and antibiotic susceptibility tests are the routine and gold standard for diagnosis of MSSA and MRSA. However, the assays are laborious and need 3–5 days to obtain a final result, which may result in a delay in prompt initiation of appropriate antimicrobial treatment. To improve the diagnosis of MSSA and MRSA, various nucleic acid amplification tests (NAATs) were developed (10). As the gold standard of molecular diagnosis, quantitative (or real-time) PCR (qPCR)-based assays are characterized by high sensitivity and specificity but require sophisticated instruments and technicians. Furthermore, a standard qPCR reaction needs about 60–90 min. Therefore, qPCR-based assays may not be optimal for the rapid diagnosis of MSSA and MRSA in resource-limited settings.

A loop-mediated isothermal amplification (LAMP) that was developed in 2000 is a robust isothermal amplification method (11). As an alternative, many LAMP assays were previously developed to detect MSSA and MRSA (12–29). However, the major weakness of the conventional LAMP method is the frequent non-specific amplification, which causes false-positive results when sequence-independent fluorescent dyes or pH-sensitive indicators are used for real-time monitoring or end-point observation (30, 31). Most of previous LAMP assays used sequence-independent fluorescent dyes (e.g., SYBR green I and SYTO9) or pH-sensitive indicators (e.g., Cresol red) (30). Furthermore, two individual assays were needed to detect both MSSA and MRSA since the conventional LAMP method lacks multiplex detection capacity. Recent development of a HiFi-LAMP method not only largely improves the specificity, sensitivity, and speed of LAMP but also enables the multiple detection of different targets in a single reaction (31–34). Similar to the qPCR methods, HiFi-LAMP method utilizes an HFman probe to improve its detection specificity and realize single-tube multiplex detection (32). In this study, we used HiFi-LAMP method to develop a novel extraction-free dual assay for simultaneous detection of MSSA and MRSA in a single tube and evaluated the assay using both prospective and retrospective clinical evaluation strategies.

## RESULTS

### Establishment of the HiFi-LAMP assay

To obtain optimal primers for detection of MSSA and MRSA, we retrieved four sets of LAMP primers targeting *nuc* gene of *S. aureus* and seven sets of LAMP primers targeting methicillin-resistant gene *mecA* from previous studies (Table S1) (21–29). The performance of these primer sets was evaluated using a LAMP system with SYTO9 as fluorescent dye as previously described (35). All reactions with the nuc- or mecA-primer sets yielded amplification curves with various time threshold (Tt) values (12–45 min) when no templates were inputted (known as non-template control) (Fig. S1), indicating the presence of non-specific amplification (false-positive). These results imply that the previous assay might have low specificity. When the same amount (3,000 copies) of DNA standard was added to the LAMP reaction, the nuc-primer set2 generated an amplification curve with the lowest Tt value (10 min) (Fig. S1a), implying faster amplification than all other nuc-primer sets (Tt: 20–30 min). Three mecA-primer sets, set3, set6, and set2, target the same *mecA* gene region and generated similar but obviously faster amplification curves (lower Tt values: 7–9 min) than all other primer sets (Tt: 10–25 min) (Table S1; Fig. S1b). In particular, the reaction with mecA-set6 had a slightly lower Tt value (7 min) than those of primer set2 and set3 (8–9 min), implying that mecA-set6 has a slightly higher amplification efficiency. Based on the results of the nuc- and mecA-primer sets together, nuc-set2 and mecA-set6 were selected and used to establish a novel dual HiFi-LAMP assay for simultaneous detection of MSSA and MRSA (Table 1). In the dual HiFi-LAMP assay, a result of positive for *nuc* gene but negative for *mecA* gene is defined as MSSA, and a result of positive for both *nuc* and *mecA* genes is defined as MRSA.

**TABLE 1:** Sequence of primers used for specific amplification of *nuc* and *mecA* genes

Genes	Primer or probe	Sequence (5′−3′)	Ref
*nuc*	nuc-Set2-F3	GAAGTGGTTCTGAAGATCCAA	(21)
	nuc-Set2-B3	CCAAGCCTTGACGAACTAA	
	nuc-Set2-FIP	AGGATGCTTTGTTTCAGGTGTCGATTGATGGTGATACGGTTA	
	nuc-Set2-BIP	AATATGGTCCTGAAGCAAGTGCGCTAAGCCACGTCCATAT	
	nuc-Set2-LF	TCTGAATGTCATTGGTTGACCT	
	nuc-Set2-LB	GAAGTCGAGTTTGACAAAGGTC	
	nuc-HFman probe	BHQ1-GAAGTCGAGTTTGACAAAGGTC-VIC	
*mecA*	mecA-Set6-F3	TGATGCTAAAGTTCAAAAGAGT	(22)
	mecA-Set6-B3	GTAATCTGGAACTTGTTGAGC	
	mecA-Set6-FIP	TGAAGGTGTGCTTACAAGTGCTAATCAACATGAAAAATGATTATGGCTC	
	mecA-Set6-BIP	TGACGTCTATCCATTTATGTATGGCAGGTTCTTTTTTATCTTCGGTTA	
	mecA-Set6-LF	TCACCTGTTTGAGGGTGGA	
	mecA-HFman probe	BHQ2-TCACCTGTTTGAGGGTGGA-Cy5	

### Specificity and sensitivity of the dual HiFi-LAMP assay

**Fig 1:**
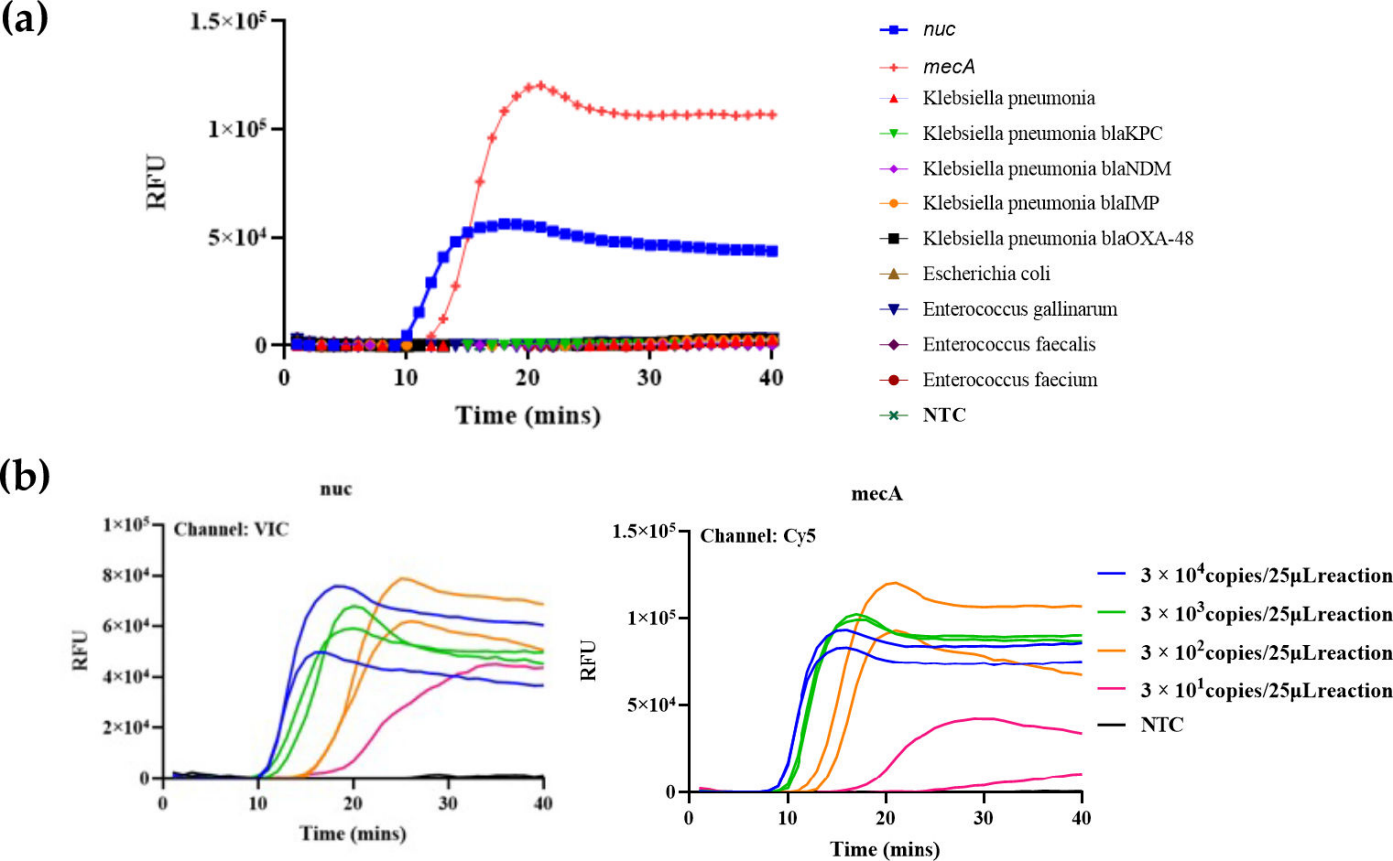
Specificity (**a**) and sensitivity (**b**) of the novel dual HiFi-LAMP assay.

We used nine common clinical bacterial isolates [*Klebsiella pneumoniae*, *Escherichia coli*, *Enterococcus gallinarum*, *Enterococcus faecalis*, *Enterococcus faecium*, *Klebsiella pneumoniae,* and four carbapenem-resistant *Klebsiella pneumoniae* (KPC, NDM, IMP, and OXA-48)] to test the specificity of the dual HiFi-LAMP assay. No amplification was generated for all these clinical isolates, indicating a great specificity (Fig. 1a). To further confirm its specificity, 20 clinical samples (urine and sputum) that were demonstrated to be negative for MSSA and MRSA by clinical microbial culture and antibiotic susceptibility tests were tested. No sample was detected positive for both *nuc* and *mecA*, supporting the great specificity of the dual HiFi-LAMP assay.

To preliminarily determine the detection ability of the novel HiFi-LAMP assay, 10-fold serial dilutions of DNA standard from 3 × 10^4^ to 3 × 10^1^ copies per microliter were used and 3 µL was added to each reaction. The detection ability of the dual assay was determined as 30 copies per 25 µL reaction, and all the positive reaction had Tt values of less than 30 min (Fig. 1b). When a single reaction system only for *nuc* or *mecA* gene was used, the detection ability increased to 3 copies per 25 µL reaction (Fig. S2).

### Limit of detection (LOD) of the dual HiFi-LAMP assay

To determine the sensitivity of the dual HiFi-LAMP assay, we tested its LOD using 16 replicates of 5-fold serial dilutions of plasmid standards (Table 2). All 16 replicate reactions (100%) were positive when 600 and more copies of *nuc* or *mecA* plasmid standards were inputted, while 14 of 16 reactions showed positive when 120 copies of *nuc* or *mecA* plasmid standards were inputted. The LOD was determined as 147 and 158 copies per 25 µL reaction for *nuc* and *mecA* genes, respectively (Table 2).

**TABLE 2:** LOD of the dual HiFi-LAMP assay for *nuc* and *mecA* genes

Template input (copies/25 µL reaction)	*nuc* gene (positive/total)	*mecA* gene (positive/total)
3,000	16/16	16/16
600	16/16	16/16
120	14/16	14/16
24	11/16	9/16
5	4/16	6/16
LOD (copies/25 µL reaction)	147.3	157.8

### Clinical evaluation

**Fig 2:**
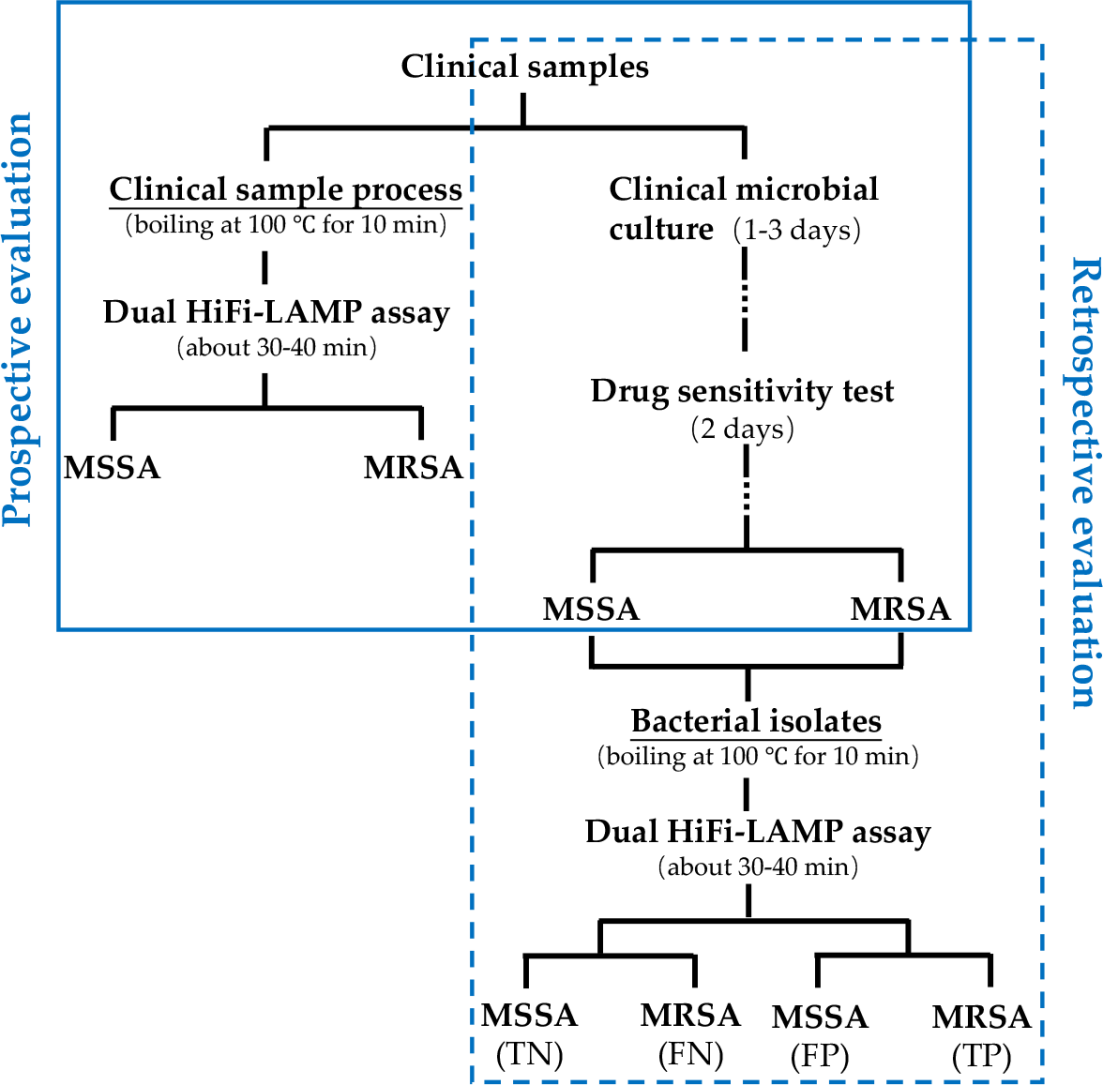
Prospective and retrospective clinical evaluation strategies. TP, TN, FP, and FN represent true positive, true negative, false positive, and false negative, respectively. In the prospective clinical evaluation, clinical samples without culture were directly subjected to the dual HiFi-LAMP assay after a simply process. In the retrospective evaluation, bacterial isolates were used.

**Fig 3:**
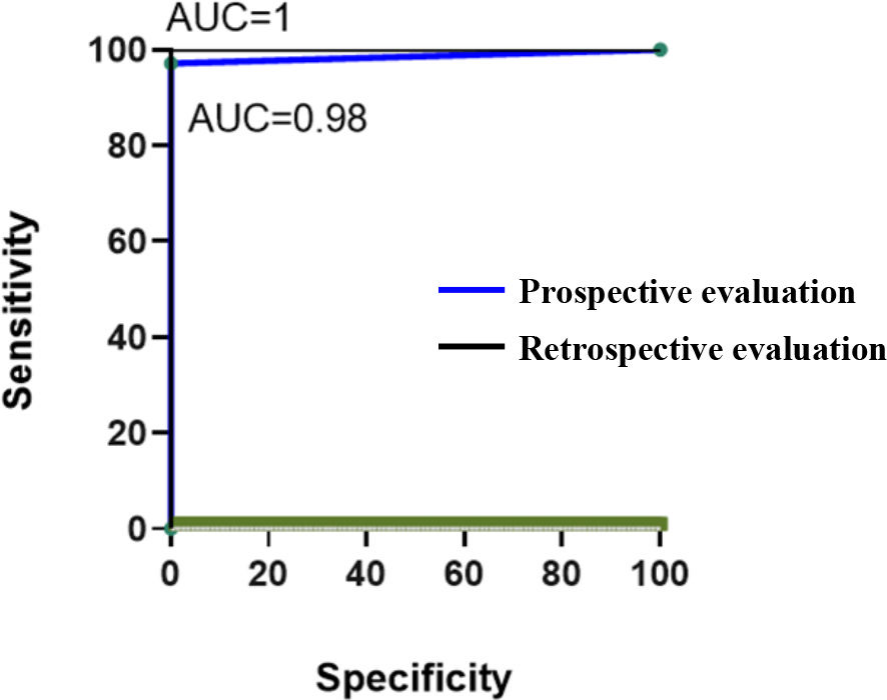
ROC curve for the novel dual HiFi-LAMP assays. AUC, area under the ROC curve.

The clinical application and POCT potential of the novel dual HiFi-LAMP assay was assessed using prospective and retrospective clinical tests with extraction-free samples (Fig. 2). In the retrospective evaluation, 107 clinical bacterial isolates were subjected to the novel dual HiFi-LAMP assay. Of them, 85 (79.4%) and 22 (20.6%) were detected as MRSA and MSSA by antibiotic susceptibility test, respectively. All 85 MRSA and 22 MSSA isolates were also detected as MRSA and MSSA by the dual HiFi-LAMP assay, respectively (Table 3). The sensitivity, specificity, ACC (total prediction accuracy), and MCC (Matthew's correlation coefficient) of the novel assay were all 100% (Table 4). In the prospective evaluation, 35 clinical samples collected from patients were included. Clinical microbial culture and antibiotic susceptibility testing identified 13 (37.1%) MRSA and 22 (62.9%) MSSA (Table 3). All 22 clinical samples with MSSA phenotype were also detected as MSSA by the novel dual HiFi-LAMP assay, whereas, of 13 samples with MRSA phenotype, 12 (92.3%) were also detected as MRSA and one was detected as MSSA by the novel assay (Table 3). The HiFi-LAMP assay showed sensitivity, specificity, ACC, and MCC of 92.3%, 100%, 97%, and 94%, respectively (Table 4), indicating that the HiFi-LAMP assay yielded results that were consistent with antibiotic susceptibility testing. Receiver operating characteristic (ROC) curve analysis showed that the dual HiFi-LAMP assay had area under the curve (AUC) of 0.98 and 1 in the prospective and retrospective clinical evaluations, respectively (Fig. 3), indicating that the novel assay has strong potential to detect MRSA and MSSA. In particular, the performance of the dual HiFi-LAMP assay was very similar between the prospective and retrospective clinical evaluations.

**TABLE 3:** Detection of clinical samples (prospective) and isolates (retrospective) by the novel dual HiFi-LAMP assay

Clinical evaluation strategies		Phenotypes by antibiotic susceptibility test			
		Prospective evaluation		Retrospective evaluation	
		MSSA	MRSA	MSSA	MRSA
Genotypes by the dual HiFi-LAMP assay	MSSA	22	1	22	0
	MRSA	0	12	0	85
	Total	22	13	22	85

**TABLE 4:** Performance of the dual HiFi-LAMP assay for clinical samples (prospective) and isolates (retrospective)

Clinical evaluation strategies	TP	TN	FP	FN	ACC (%)	Sp (%)	Se (%)	MCC (%)
Prospective evaluation	12	22	0	1	97	100	92.3	94.0
Retrospective evaluation	85	22	0	0	100	100	100	100

^*a*^ Sp: specificity; Se: sensitivity; ACC: total prediction accuracy; MCC: Matthew’s correlation coefficient.

**Fig 4:**
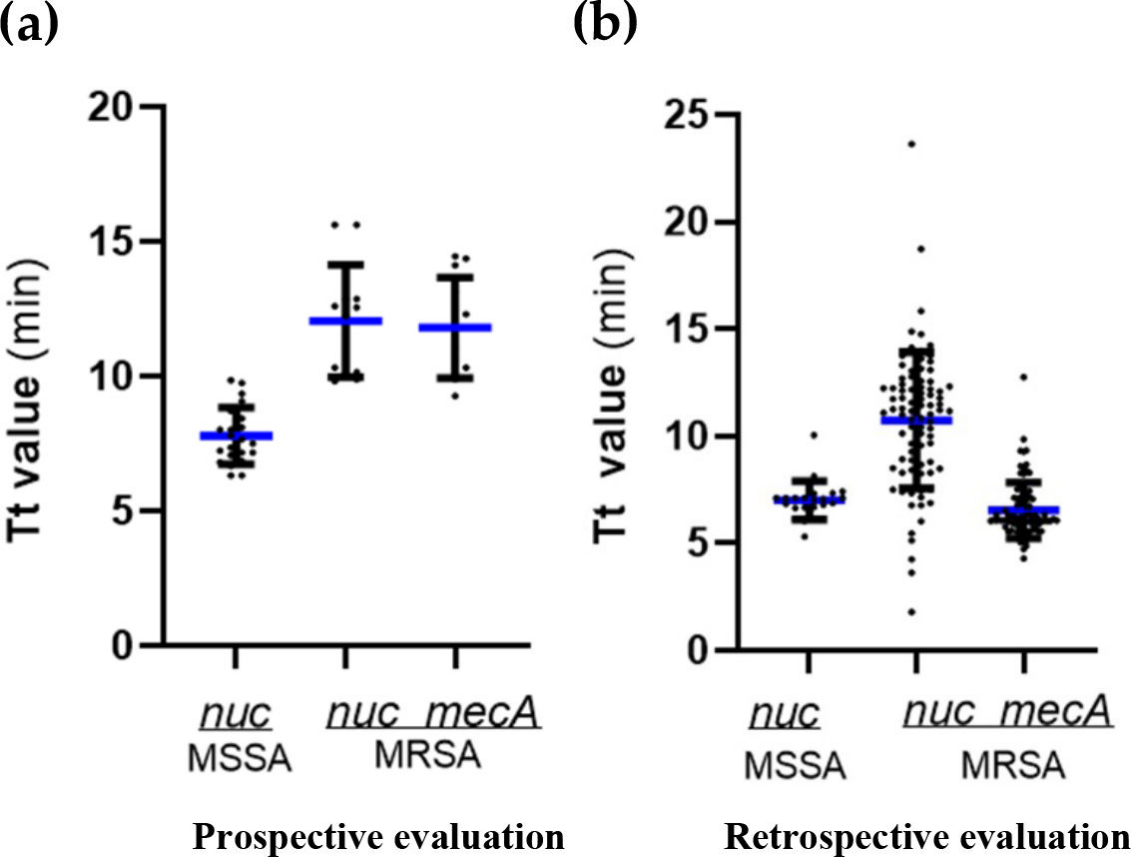
Tt values of the novel HiFi-LAMP assay for detection of MSSA and MRSA in prospective (**a**) and retrospective (**b**) clinical evaluations.

In clinical evaluations, all MSSA positive samples by the HiFi-LAMP assay had Tt values of less than 10 min (*nuc* gene), and all MRSA positive samples had Tt values of less than 15 min for *mecA* gene and less than 20 min for *nuc* gene except one with a Tt value of less than 25 min (Fig. 4). These results indicated that the novel dual HiFi-LAMP assay is very fast and can be completed within 30 min.

To figure out the reason for the discrepancy between phenotype (MRSA) and genotype (MSSA) in one clinical sample (36), we further tested the original clinical sample and its clinical isolate from an antibiotic susceptibility testing using a commercial qPCR kit (Liferiver, Shanghai, China). The qPCR assay also detected the clinical sample as MSSA, whereas its clinical isolate was detected as MRSA. These results might imply that this sample contains a very low abundance of the *mecA* gene or that the culture was mixed when tested using antibiotic susceptibility test methods.

## DISCUSSION

*S. aureus* is a Gram-positive, spherical-shaped bacterium. It can be found on the healthy skin of about 30% of the population and does not cause obvious disease symptoms (1, 3, 5, 37). However, it can result in serious diseases once entering the internal tissues and bloodstream. MRSA emerged in the United Kingdom in 1961 and spread worldwide (2, 6, 7). The resistance mechanism of MRSA was mainly ascribed to a penicillin-binding protein 2a (PBP2a) that has a very low affinity for β-lactams (38, 39). PBP2a is encoded by *mecA* gene that is located in the staphylococcal cassette chromosome mec SCCmec. MRSA strains are resistant to a wide range of antibiotics, including methicillin, penicillin, oxacillin, cloxacillin, cefazolin, cefoxitin, and so on (40). MRSA can be transmitted by contact with infected people and was often associated with HAIs and CAIs (2, 7). Therefore, it has been listed as a “priority pathogen” by the World Health Organization (6).

Accurate and rapid point-of-care (POC) detection of MRSA plays a critical role in the prevention and control of HAIs and CAIs, as well as clinical management and treatment of MRSA infections (8, 9). Some LAMP-based assays have been previously developed for POC detection of MRSA and/or MSSA (12–22, 24). However, most of these assays were limited by low detection specificity caused by frequent non-specific amplification of LAMP method and/or lack of a single-pot multiplex detection of both MRSA and MSSA. As validated by qPCR methods, the use of probes in NAATs can significantly improve the detection specificity and enable multiplex detection. To improve the specificity of LAMP assays, various probe-based LAMP methods have been developed (31). Recently, we developed an HFman probe-based HiFi-LAMP method that uses a small amount of additional high-fidelity DNA polymerase to recognize and cleave the HFman probe to release fluorescent signal (31, 32). The HiFi-LAMP method realizes highly specific, sensitive, and faster multiplex detection of different targets in a single reaction (32, 33, 34, 41, 42). Using the new method, we developed a novel dual HiFi-LAMP assay for simultaneous detection of both MRSA and MSSA.

The detection abilities of the single and dual HiFi-LAMP assays can reach 3 and 30 copies of both *nuc* and *mecA* genes per 25 µL reaction, respectively. The LOD of the dual HiFi-LAMP assay was 147 and 158 copies of *nuc* and *mecA* genes per 25 µL reaction. The great specificity of the novel assay was validated by nine common clinical bacterial isolates and 20 clinical samples infected by non-MSSA and non-MRSA bacteria.

The novel extraction-free dual HiFi-LAMP assay showed 100% of both sensitivity and specificity for detection of MRSA and MSSA in a retrospective clinical evaluation with 107 clinical *S. aureus* isolates. A prospective clinical evaluation with 35 clinical samples revealed 92.3% detection sensitivity and 100% specificity of the extraction-free dual HiFi-LAMP assay. The only failure in detection of MRSA in one clinical sample by both the novel assay and the commercial qPCR assay might be ascribed to very low abundance of *mecA* gene in the sample or potential contamination during antibiotic susceptibility test.

The novel dual HiFi-LAMP assay was characterized by a faster amplification generally within 30 min. All positive amplification signal by the novel assay appeared within 20 min regardless of prospective or retrospective detections (except one for *nuc* gene within 25 min in the retrospective detection). Together with about 10 min of rapid sample processing, the extraction-free dual HiFi-LAMP assay can be completed within 40 min, significantly shorter than about 3–5 days by the traditional clinical microbial culture and antibiotic susceptibility testing. Therefore, the use of extraction-free dual HiFi-LAMP assay can promote precise diagnosis and treatment of MRSA infections and can reduce empirical therapy prior to antibiotic susceptibility testing.

In summary, we developed a sensitive, specific, and fast extraction-free dual HiFi-LAMP assay to detect MRSA and MSSA. The assay has LOD of 147 and 158 copies of *nuc* and *mecA* genes per 25 µL reaction and a short sample-to-answer times of about 40 min. Accompanied with the availability of various portable (isothermal) fluorimeters for real-time monitoring in clinic and community settings, the novel extraction-free dual HiFi-LAMP assay may be very helpful not only in promoting precise diagnosis and treatment of MRSA infections in hospitals but also in facilitating MRSA surveillance in hospital and community settings.

## MATERIALS AND METHODS

### Preparation of plasmid standards

Plasmids containing *nuc* gene (GenBank: DQ3996781.1) of *S. aureus* and the *mecA* gene (GenBank: AB221123.1) of MRSA were synthesized by Shanghai Sangon BioEngineering Co., LTD. Plasmid concentrations were measured by NanoDrop One/OneC trace UV-Vis Spectrophotometer (Thermo Fisher Scientific). The copy number of plasmid was calculated using the following formula: DNA copy number/mL = [DNA concentration (g/mL)/(DNA length × 660)] × 6.022 × 10^23^.

### Selection of primers and probes

Four and seven sets of LAMP primers targeting to *nuc* and *mecA* genes, respectively, were previously reported (Table S1) (21–29). We synthesized all these primers (by Shanghai Sangon Biotechnology Co., LTD) and performed comparative experiments to select the optimal LAMP primer sets for detection of MSSA and MRSA as previously described (35). Each set of primers was performed in a 25 µL LAMP reaction containing 1 × isothermal amplification buffer, 8 mM MgSO4, 1.8 mM dNTP, 8 U Bst 4.0 DNA polymerase (Haigene, China), 0.15 U high-fidelity DNA polymerase (New England Biolabs, Inc., MA, USA), 0.4 mM SYTO9 (Life Technologies, CA, USA), 0.1 µM F3 and B3 each, 1.0 µM FIP and BIP each, and 0.6 µM LB and/or LF each. A total of 3,000 copies of DNA standard were added in each reaction. The reactions were performed at 64°C for 50 min using a Bio-Rad CFX96 real-time PCR detection system.

The primer set generating the lowest time threshold (Tt) was selected (Fig. S1). As a result, the primer set2 for *nuc* gene and the primer set6 for *mecA* gene were selected for developing dual HiFi-LAMP assay for detection MSSA and MRSA (Table 1). The nuc-HFman probe was further synthesized by labeling a VIC fluorophore and BHQ1 quencher at the 3′ and 5′ ends of nuc-LB primer, and mecA-HFman probe was synthesized by labeling a CY5 fluorophore and BHQ2 quencher at the 3′ and 5′ ends of mecA-LF primer, respectively.

### Dual HiFi-LAMP reaction system

The dual HiFi-LAMP mixture was same with above LAMP reaction system except replacing 0.4 mM SYTO9 with 0.3 µM both HFman probes and reducing the concentrations of nuc-LB and mecA-LF from 0.6 μM to 0.3 µM. The reactions were also performed at 64°C for 50 min using a Bio-Rad CFX96 real-time PCR detection system, and the fluorescent signals emitted by VIC and Cy5 were measured each minute.

### Sensitivity and specificity

The detection ability of the HiFi-LAMP assay was preliminarily measured with 10-fold serial dilutions of DNA standard ranging from 3.0 × 10^4^ to 3.0 × 10^1^ copies per 25 µL reaction. The specificity of the HiFi-LAMP assay was assessed using nine common clinical bacterial isolates, including *Klebsiella pneumoniae*, *Escherichia coli*, *Enterococcus gallinarum*, *Enterococcus faecalis*, *Enterococcus faecium*, *Klebsiella pneumoniae*, and four carbapenem-resistant *Klebsiella pneumoniae* (KPC, NDM, IMP, and OXA-48). These isolates were obtained from clinical samples after clinical microbial culture and antibiotic susceptibility test.

### Limit of detection (LOD)

To determine the LOD of the HiFi-LAMP assay, 25 µL reactions were performed with 5-fold serial dilutions of the plasmid standard from 3,000, 600, 120, 24, to 5 copies. Sixteen replicates were done for each template concentration. The LOD was determined as the 95% probability of obtaining a true positive result using probit regression analysis implemented in the SPSS 17.0 software.

### Clinical evaluation of the novel dual HiFi-LAMP assay

We used prospective and retrospective clinical evaluation strategies to evaluate the novel dual HiFi-LAMP assay (Fig. 2). The retrospective clinical evaluation was performed using 107 clinical *S. aureus* isolates. These isolates were identified as 85 MRSA and 22 MSSA by clinical microbial culture and antibiotic susceptibility testing from March to September 2023.

In the prospective clinical evaluation, clinical samples (whole blood, secretions, sputum, or puncture fluid) collected from various patients were divided into two aliquots. One aliquot was subjected to routine clinical microbial culture and antibiotic susceptibility testing according to different sample types. Another one was directly subjected to the HiFi-LAMP assay. Before the HiFi-LAMP assay, appropriate amounts of secretion, sputum, or puncture fluid were mixed with 200 µL deionized water, boiled at 100°C for 10 min, and then centrifuged at 10,000 rpm for 5 min. About 10 mL whole blood was added into 40 mL blood medium, and then 0.5 mL of the mix was boiled at 100°C for 10 min, and then centrifuged at 10,000 rpm for 5 min. Five microliters of the supernatant was used in the HiFi-LAMP assay and the remaining supernatant was frozen at −80°C for further use.

The clinical evaluations were conducted in accordance with the Declaration of Helsinki and approved by the Ethics Committee of Taizhou Fourth People's Hospital (No. 2023-EC/TZFH-23). Oral informed consents were obtained from the patients recruited in the prospective evaluation. The detailed information of involved patients in prospective and retrospective evaluation tests are available in Tables S2 and S3.

### Statistical analysis

The performance of the HiFi-LAMP assay was determined by measuring the sensitivity (SE), specificity (SP), total prediction accuracy (ACC), and Matthew's correlation coefficient (MCC), which were calculated by the following equations. The results of clinical microbial culture and antibiotic susceptibility testing were considered as gold standard, and MRSA and MSSA phenotypes are defined as positive and negative, respectively.


$$ACC=\frac{TP+TN}{TP+TN+FP+FN}$$

$$Sp=\frac{TN}{TN+FP}$$

$$Se=\frac{TP}{TP+FN}$$

$$MCC=\frac{TP\times TN-FP\times FN}{\sqrt{\left(TP+FP\right)\times \left(TN+FN\right)\times \left(TP+FN\right)\times \left(TN+FP\right)}}$$

In the above equations, TP (true positive) and FN (false negative) are defined as MRSA phenotype to be determined as MRSA and MSSA, and TN (true negative) and FP (false positive) are defined as MSSA phenotype to be determined as MSSA and MRSA by the HiFi-LAMP assay, respectively.

## SUPPLEMENTAL MATERIAL

Supplementary material spectrum.04133-23-s0001.docx Supplementary Tables S1 to S3 and Figures S1 and S2.

ASM does not own the copyrights to Supplemental Material that may be linked to, or accessed through, an article. The authors have granted ASM a non-exclusive, world-wide license to publish the Supplemental Material files. Please contact the corresponding author directly for reuse.
