# Supplementary material for: A novel extraction-free dual HiFi-LAMP assay for detection of methicillin-sensitive and methicillin-resistant Staphylococcus aureus
Source: Microbiol Spectr. 2024 Feb 20;12(4):e04133-23. doi: 10.1128/spectrum.04133-23 (PMC10986577; doi:10.1128/spectrum.04133-23)
Supplement: Supplementary material — Supplementary Tables S1 to S3 and Figures S1 and S2. [file spectrum.04133-23-s0001.docx]

Supplementary Materials

**for**

A novel extraction-free dual HiFi-LAMP assay for detection of methicillin-sensitive and methicillin-resistant *Staphylococcus aureus*

Xiuli Zhao^1^, Yi Zeng^2^, Beibei Yan^1^, Yanping Liu^1^, Yueqin Qian^1^, Aiping Zhu^1^, Yongjuan Zhao^2^, Xiaoling Zhang^2^, Chiyu Zhang^2,^ *, Zhenzhou Wan^1,^ *

^1^ Medical Laboratory of Taizhou Fourth People’s Hospital, Taizhou 225300 China;

^2^ Shanghai Public Health Clinical Center, Fudan University, Shanghai 201508, China;

***** Correspondence: chiyu_zhang1999@163.com (C.Z.); or wanlv@126.com (Z.W.)

**Table S1.** Several sets of primers of *S. aureus* and MRSA.

| **Primer sets** | **Primer name** | **Sequence** (5′-3′) | **Length**  (nt) | **Gene location** (nt) | **Refs.** |
| --- | --- | --- | --- | --- | --- |
| Nuc-set1 | F3 | ATGCAAAGAAAATTGAAGTCGA | 22 | 452-683 | [1] |
|  | B3 | GCGTTGTCTTCGCTCCAAAT | 20 |  |  |
|  | FIP | CGTTTACCATTTTTCCATCAGCATAGTTTGACAAAGGTCAAAGAACT | 47 |  |  |
|  | BIP | TCAAGGCTTGGCTAAAGTTGCTTATTTTCGCTTGTGCTTCACTT | 44 |  |  |
|  | LF | TACGCTAAGCCACGTCCATA | 20 |  |  |
|  | LB | CCTAACAATACACATGAACAAC | 22 |  |  |
| Nuc-set2 | F3 | GAAGTGGTTCTGAAGATCCAA | 21 | 218-574 | [2] |
|  | B3 | CCAAGCCTTGACGAACTAA | 19 |  |  |
|  | FIP | AGGATGCTTTGTTTCAGGTGTCGATTGATGGTGATACGGTTA | 42 |  |  |
|  | BIP | AATATGGTCCTGAAGCAAGTGCGCTAAGCCACGTCCATAT | 40 |  |  |
|  | LF | TCTGAATGTCATTGGTTGACCT | 22 |  |  |
|  | LB | GAAGTCGAGTTTGACAAAGGTC | 22 |  |  |
| Nuc-set3 | F3 | AACAGTATATAGTGCAACTTCAA | 23 | 237-484 | [3] |
|  | B3 | CTTTGTCAAACTCGACTTCAA | 21 |  |  |
|  | FIP | ATGTCATTGGTTGACCTTTGTACATAAATTACATAAAGAACCTGCGA | 47 |  |  |
|  | BIP | GTTGATACACCTGAAACAAAGCATCATTTTTTTCGTAAATGCACTTGC | 48 |  |  |
|  | LF | GTATCACCATCAATCGCTTT | 20 |  |  |
|  | LB | GGTGTAGAGAAATATGGTCC | 20 |  |  |
| Nuc-set4 | F3 | AAATGCAAAGAAAATTGAAGTCG | 23 | 450-682 | [4] |
|  | B3 | CGTTGTCTTCGCTCCAAAT | 19 |  |  |
|  | FIP | CGTTTACCATTTTTCCATCAGCATAAGTTTGACAAAGGTCAAAGAACT | 48 |  |  |
|  | BIP | GTCAAGGCTTGGCTAAAGTTGCTTATTCGCTTGTGCTTCACTT | 43 |  |  |
|  | LF | GCTAAGCCACGTCCAT | 16 |  |  |
|  | LB | CCTAACAATACACATGAAC | 19 |  |  |
| mecA-set1 | F3 | GATGAATATTTAAGWGATTTCGC | 23 | 751-965 | [5] |
|  | B3 | TGGAGCTTTTTATCGTAAAGTT | 22 |  |  |
|  | FIP | ACCTAATAGATGTGAAGTCGCTTTTTTCATCTTACAACTAATGAAACAGAA | 51 |  |  |
|  | BIP | TATGTTGGTCCCATTAACTCTGAAGTTCCCTTTTTACCAATAACTGCA | 48 |  |  |
|  | LF | TTCTAGAGGATAGTTACGACT | 21 |  |  |
|  | LB | CAAAAAGAATATAAAGGCTATAA | 23 |  |  |
| mecA-set2 | F3 | TGATGCTAAAGTTCAAAAGAGT | 22 | 1074-1284 | [4] |
|  | B3 | TGTAATCTGGAACTTGTTGAG | 21 |  |  |
|  | FIP | TGAAGGTGTGCTTACAAGTGCTAATAAATGATTATGGCTCAGGTACT | 47 |  |  |
|  | BIP | TGACGTCTATCCATTTATGTATGGCGAGGTTCTTTTTTATCTTCGGTTA | 49 |  |  |
|  | LF | GTTTGAGGGTGGATAGC | 17 |  |  |
| mecA-set3 | F3 | TGATGCTAAAGTTCAAAAGAGT | 22 | 1074-1283 | [6] |
|  | B3 | GTAATCTGGAACTTGTTGACC | 21 |  |  |
|  | FIP | AGGTGTGCTTACAAGTGCTAATAATCAACATGAAAAATGATTATGGCT | 48 |  |  |
|  | BIP | TGACGTCTATCCATTTATGTATGGCAGGTTCTTTTTTATCTTCGGTTA | 48 |  |  |
|  | LF | TGAGGGTGGATAGCAGTACC | 20 |  |  |
|  | LB | TGAGTAACGAAGAATAT | 17 |  |  |
| mecA-set4 | F3 | GGCTCAGGTACTGCTATC | 18 | 1123-1340 | [7] |
|  | B3 | TTGTTATTTAACCCAATCATTGC | 23 |  |  |
|  | FIP | ATGCCATACATAAATGGATAGACGTCAAACAGGTGAATTATTAGCACTT | 49 |  |  |
|  | BIP | CCGAAGATAAAAAAGAACCTCTGCTTTTTTGAGTTGAACCTGGTG | 45 |  |  |
|  | LF | CATATGAAGGTGTGCTTAC | 19 |  |  |
|  | LB | CAAGTTCCAGATTACAACTT | 20 |  |  |
| mecA-set5 | F3 | AAGATGGCAAAGATATTCAACT | 22 | 1046-1260 | [8] |
|  | B3 | AGGTTCTTTTTTATCTTCGGTTA | 23 |  |  |
|  | FIP | GTGGATAGCAGTACCTGAGCCTTGATGCTAAAGTTCAAAAGAGT | 44 |  |  |
|  | BIP | CCTCAAACAGGTGAATTATTAGCACCTTCGTTACTCATGCCATAC | 45 |  |  |
|  | LF | TAATCATTTTTCATGTTG | 18 |  |  |
|  | LB | TGTAAGCACACCTTCATATGACGT | 24 |  |  |
| mecA-set6 | F3 | TGATGCTAAAGTTCAAAAGAGT | 22 | 1074-1283 | [3] |
|  | B3 | GTAATCTGGAACTTGTTGAGC | 21 |  |  |
|  | FIP | TGAAGGTGTGCTTACAAGTGCTAATCAACATGAAAAATGATTATGGCTC | 49 |  |  |
|  | BIP | TGACGTCTATCCATTTATGTATGGCAGGTTCTTTTTTATCTTCGGTTA | 48 |  |  |
|  | LF | TCACCTGTTTGAGGGTGGA | 19 |  |  |
| mecA-set7 | F3 | GGTACAAGATGATACCTTCGTT | 22 | 705-1139 | [9] |
|  | B3 | ATAGCAGTACCTGAGCCAT | 19 |  |  |
|  | FIP | TCTTCAGAGTTAATGGGACCAAACAGAAAGTCGTAACTATCCTC | 44 |  |  |
|  | BIP | AAGCTCCAACATGAAGATGGCTTGTATGTGCGATTGTATTGC | 42 |  |  |
|  | LF | ACCTAATAGATGTGAAGTCGCT | 22 |  |  |
|  | LB | CGTGTCACAATCGTTGACG | 19 |  |  |

**References**

[1] Wang Y, Li H, Wang Y, Zhang L, Xu J, Ye C. Loop-Mediated Isothermal Amplification Label-Based Gold Nanoparticles Lateral Flow Biosensor for Detection of Enterococcus faecalis and Staphylococcus aureus. Frontiers in microbiology. 2017;8:192. <https://doi.org/10.3389/fmicb.2017.00192>

[2] Sheet OH, Grabowski NT, Klein G, Abdulmawjood A. Development and validation of a loop mediated isothermal amplification (LAMP) assay for the detection of Staphylococcus aureus in bovine mastitis milk samples. Molecular and cellular probes. 2016;30:320-5. <https://doi.org/10.1016/j.mcp.2016.08.001>

[3] Wang XR, Wu LF, Wang Y, Ma YY, Chen FH, Ou HL. Rapid detection of Staphylococcus aureus by loop-mediated isothermal amplification. Applied biochemistry and biotechnology. 2015;175:882-91. <https://doi.org/10.1007/s12010-014-1328-x>

[4] Jiang L, Li X, Gu R, Mu D. Nanoparticles-Based Biosensor Coupled with Multiplex Loop-Mediated Isothermal Amplification for Detection of Staphylococcus aureus and Identification of Methicillin-Resistant S. aureus. Infection and drug resistance. 2020;13:1251-62. <https://doi.org/10.2147/idr.s243881>

[5] Choopara I, Suea-Ngam A, Teethaisong Y, Howes PD, Schmelcher M, Leelahavanichkul A, et al. Fluorometric Paper-Based, Loop-Mediated Isothermal Amplification Devices for Quantitative Point-of-Care Detection of Methicillin-Resistant Staphylococcus aureus (MRSA). ACS sensors. 2021;6:742-51. <https://doi.org/10.1021/acssensors.0c01405>

[6] Kim SG, Choi GW, Choi WS, Lim CS, Jang WS, Bae JH. Feasibility of Loop-Mediated Isothermal Amplification for Rapid Detection of Methicillin-Susceptible and Methicillin-Resistant Staphylococcus aureus in Tissue Samples. Clinics in orthopedic surgery. 2022;14:466-73. <https://doi.org/10.4055/cios21277>

[7] Chen X, Ma K, Yi X, Xiong L, Wang Y, Li S. The rapid and visual detection of methicillin-susceptible and methicillin-resistant Staphylococcus aureus using multiplex loop-mediated isothermal amplification linked to a nanoparticle-based lateral flow biosensor. Antimicrobial resistance and infection control. 2020;9:111. <https://doi.org/10.1186/s13756-020-00774-x>

[8] Metwally L, Gomaa N, Hassan R. Detection of methicillin-resistant Staphylococcus aureus directly by loop-mediated isothermal amplification and direct cefoxitin disk diffusion tests. Eastern Mediterranean health journal = La revue de sante de la Mediterranee orientale = al-Majallah al-sihhiyah li-sharq al-mutawassit. 2014;20:273-9.

[9] Sudhaharan S, Vanjari L, Mamidi N, Ede N, Vemu L. Evaluation of LAMP Assay Using Phenotypic Tests and Conventional PCR for Detection of nuc and mecA genes Among Clinical Isolates of Staphylococcus spp. Journal of clinical and diagnostic research : JCDR. 2015;9:Dc06-9. <https://doi.org/10.7860/jcdr/2015/13962.6315>

**Table S2.** Patients’ information in posterior clinical evaluation.

| **Patients’ information** | | | | **Drug sensitivity test** | | **HiFi-LAMP assay** | |
| --- | --- | --- | --- | --- | --- | --- | --- |
| **Clinical diagnosis** | **No.** | **Gender** | **Age**(years) | **MRSA** | **MSSA** | **MRSA** | **MSSA** |
| **Soft tissue infections** | 1 | M | 69 | + | - | + | - |
|  | 2 | M | 15 | + | - | + | - |
|  | 3 | M | 69 | + | - | + | - |
|  | 4 | M | 80 | + | - | + | - |
|  | 5 | M | 80 | + | - | + | - |
|  | 6 | M | 79 | + | - | + | - |
| **Skin infections** | 7 | F | 69 | + | - | + | - |
|  | 8 | F | 80 | + | - | + | - |
|  | 9 | M | 78 | + | - | + | - |
| **Bacteraemia, lacunar cerebral infarction** | 10 | M | 74 | + | - | + | - |
|  | 11 | M | 74 | + | - | + | - |
| **Lung infections** | 12 | M | 76 | + | - | + | - |
|  | 13 | M | 70 | + | - | + | - |
|  | 14 | M | 81 | + | - | + | - |
| **Hemiplegia** | 15 | F | 72 | + | - | + | - |
|  | 16 | M | 40 | + | - | + | - |
|  | 17 | F | 66 | + | - | + | - |
|  | 18 | M | 57 | + | - | + | - |
|  | 19 | M | 78 | + | - | + | - |
|  | 20 | F | 75 | + | - | + | - |
|  | 21 | M | 76 | + | - | + | - |
| **Severe pneumonia** | 22 | F | 57 | + | - | + | - |
|  | 23 | M | 1 month | + | - | + | - |
|  | 24 | M | 4 | + | - | + | - |
|  | 25 | F | 1 month | + | - | + | - |
|  | 26 | M | 78 | + | - | + | - |
|  | 27 | F | 89 | + | - | + | - |
|  | 28 | F | 76 | + | - | + | - |
|  | 29 | M | 77 | + | - | + | - |
|  | 30 | F | 79 | + | - | + | - |
|  | 31 | F | 88 | + | - | + | - |
| **Vasculitis of the lower extremities** | 32 | M | 66 | + | - | + | - |
| **Coronary heart disease** | 33 | M | 89 | + | - | + | - |
| **Damage** | 34 | M | 70 | + | - | + | - |
| **Health check-ups** | 35 | M | 71 | + | - | + | - |
| **Knee pain** | 36 | F | 39 | + | - | + | - |
| **Multiple burns** | 37 | M | 29 | + | - | + | - |
| **Post-traumatic wound infection** | 38 | M | 67 | + | - | + | - |
| **Post-traumatic wound infection** | 39 | F | 58 | + | - | + | - |
|  | 40 | F | 18 | + | - | + | - |
| **Acute exacerbation of chronic obstructive pulmonary disease** | 41 | M | 76 | + | - | + | - |
|  | 42 | M | 65 | + | - | + | - |
| **Back mass** | 43 | F | 65 | + | - | + | - |
|  | 44 | M | 67 | + | - | + | - |
| **Traumatic epidural hemorrhage** | 45 | M | 56 | + | - | + | - |
|  | 46 | F | 70 | + | - | + | - |
|  | 47 | M | 78 | + | - | + | - |
|  | 48 | M | 85 | + | - | + | - |
|  | 49 | M | 79 | + | - | + | - |
|  | 50 | F | 56 | + | - | + | - |
|  | 51 | M | 63 | + | - | + | - |
| **Cerebral infarction** | 52 | M | 78 | + | - | + | - |
|  | 53 | M | 82 | + | - | + | - |
|  | 54 | M | 78 | + | - | + | - |
| **Malignancy of the esophagus** | 55 | M | 69 | + | - | + | - |
|  | 56 | M | 76 | + | - | + | - |
| **Biliary tract infections** | 57 | M | 68 | + | - | + | - |
|  | 58 | M | 75 | + | - | + | - |
| **Diabetic ketoacidosis/diabetic foot** | 59 | M | 57 | + | - | + | - |
|  | 60 | M | 78 | + | - | + | - |
|  | 61 | F | 55 | + | - | + | - |
|  | 62 | F | 48 | + | - | + | - |
| **Sepsis** | 63 | F | 75 | + | - | + | - |
|  | 64 | M | 78 | + | - | + | - |
| **Infectious fever** | 65 | F | 84 | + | - | + | - |
|  | 66 | M | 6 | + | - | + | - |
| **ileus** | 67 | M | 79 | + | - | + | - |
| **Open head injury/intracranial infection** | 68 | F | 70 | + | - | + | - |
|  | 69 | M | 75 | + | - | + | - |
|  | 70 | F | 68 | + | - | + | - |
| **Basilar artery occlusion** | 71 | M | 54 | + | - | + | - |
| **Hypertension grade 3 (very high risk)** | 72 | M | 54 | + | - | + | - |
| **Hemorrhage in the thalamus** | 73 | M | 73 | + | - | + | - |
| **After left ureteral stent implantation** | 74 | F | 59 | + | - | + | - |
| **Bronchiectasis with infection** | 75 | M | 67 | + | - | + | - |
| **Organophosphate poisoning** | 76 | M | 86 | + | - | + | - |
| **Cerebellar hemorrhage** | 77 | M | 58 | + | - | + | - |
| **Open tibial fracture** | 78 | M | 53 | + | - | + | - |
| **Hyperkalemia** | 79 | M | 55 | + | - | + | - |
| **Basal nodal hemorrhage** | 80 | F | 54 | + | - | + | - |
| **Chronic renal failure (renal insufficiency) with infection** | 81 | F | 73 | + | - | + | - |
| **Ureteral space-occupying lesions** | 82 | F | 59 | + | - | + | - |
| **Tuberculous pleurisy** | 83 | F | 43 | + | - | + | - |
| **Difficult to extubate after tracheostomy** | 84 | M | 72 | + | - | + | - |
| **Severe anemia** | 85 | F | 58 | + | - | + | - |
| **Septicemia** | 86 | F | 75 | - | + | - | + |
| **Acute left-sided heart failure** | 87 | F | 68 | - | + | - | + |
| **Chronic renal failure (renal insufficiency) with infection** | 88 | M | 42 | - | + | - | + |
| **Traumatic subdural hemorrhage** | 89 | M | 74 | - | + | - | + |
| **Polymyalgia rheumatica** | 90 | M | 68 | - | + | - | + |
| **Paraplegia [paralysis of both lower limbs]** | 91 | M | 54 | - | + | - | + |
| **Pancreatic head malignancy stage IV** | 92 | F | 43 | - | + | - | + |
| **Basal ganglia hemorrhage** | 93 | F | 66 | - | + | - | + |
| **Finger flexor tendon adhesions** | 94 | M | 25 | - | + | - | + |
| **Gastric malignancy** | 95 | M | 76 | - | + | - | + |
| **Coronary atherosclerotic heart disease** | 96 | M | 38 | - | + | - | + |
| **Bronchopneumonia** | 97 | M | 6 | - | + | - | + |
| **Brain herniation** | 98 | M | 61 | - | + | - | + |
| **Multiple boils** | 99 | F | 77 | - | + | - | + |
| **Urethral mons** | 100 | F | 73 | - | + | - | + |
| **Paraplegia [paralysis of both lower limbs]** | 101 | M | 54 | - | + | - | + |
| **Infection of the incorpoisation after surgery** | 102 | M | 13 | - | + | - | + |
| **Deep vein thrombosis of the upper extremities** | 103 | F | 29 | - | + | - | + |
| **Supportive care for malignancy** | 104 | F | 74 | - | + | - | + |
| **Fracture of the lower end of the tibia and fibula** | 105 | F | 72 | - | + | - | + |
| **Neonatal pneumonia** | 106 | M | 20 days | - | + | - | + |
| **Traumatic subdural hemorrhage** | 107 | F | 55 | - | + | - | + |

**Note:** M: male; F: female; +: positive; -: negative.

**Table S3.** Patients’ information in prior clinical evaluation.

| **Patients’ information** | | | | **Drug sensitivity test** | | **HiFi-LAMP assay** | |
| --- | --- | --- | --- | --- | --- | --- | --- |
| **Clinical diagnosis** | **No.** | **Gender** | **Age**(years) | **MRSA** | **MSSA** | **MRSA** | **MSSA** |
| **Soft tissue infections of the lower extremities** | 1 | M | 69 | + | - | + | - |
|  | 2 | M | 69 | + | - | + | - |
|  | 3 | M | 80 | + | - | + | - |
|  | 4 | M | 80 | + | - | + | - |
|  | 5 | M | 49 | + | - | + | - |
| **Skin infections** | 6 | F | 80 | + | - | + | - |
|  | 7 | F | 69 | + | - | - | - |
| **Vasculitis of the lower extremities** | 8 | M | 66 | + | - | + | - |
| **Bacteraemia, lacunar cerebral infarction** | 9 | M | 74 | + | - | + | - |
|  | 10 | M | 74 | + | - | + | - |
| **coronary heart disease** | 11 | M | 89 | + | - | + | - |
| **damage** | 12 | M | 70 | + | - | + | - |
| **Health check-ups** | 13 | M | 71 | + | - | + | - |
| **septicemia** | 14 | F | 75 | - | + | - | + |
| **Acute left-sided heart failure** | 15 | F | 68 | - | + | - | + |
| **Chronic renal failure (renal insufficiency) with infection** | 16 | M | 42 | - | + | - | + |
| **Traumatic subdural hemorrhage** | 17 | M | 74 | - | + | - | + |
| **Polymyalgia rheumatica** | 18 | M | 68 | - | + | - | + |
| **Paraplegia [paralysis of both lower limbs]** | 19 | M | 54 | - | + | - | + |
| **Pancreatic head malignancy stage IV** | 20 | F | 43 | - | + | - | + |
| **Basal ganglia hemorrhage** | 21 | F | 66 | - | + | - | + |
| **Finger flexor tendon adhesions** | 22 | M | 25 | - | + | - | + |
| **Gastric malignancy** | 23 | M | 76 | - | + | - | + |
| **Coronary atherosclerotic heart disease** | 24 | M | 38 | - | + | - | + |
| **Bronchopneumonia** | 25 | M | 6 | - | + | - | + |
| **Brain herniation** | 26 | M | 61 | - | + | - | + |
| **Multiple boils** | 27 | F | 77 | - | + | - | + |
| **Urethral mons** | 28 | F | 73 | - | + | - | + |
| **Paraplegia [paralysis of both lower limbs]** | 29 | M | 54 | - | + | - | + |
| **Infection of the incorpoisation after surgery** | 30 | M | 13 | - | + | - | + |
| **Deep vein thrombosis of the upper extremities** | 31 | F | 29 | - | + | - | + |
| **Supportive care for malignancy** | 32 | F | 74 | - | + | - | + |
| **Fracture of the lower end of the tibia and fibula** | 33 | F | 72 | - | + | - | + |
| **Neonatal pneumonia** | 34 | M | 20 days | - | + | - | + |
| **Traumatic subdural hemorrhage** | 35 | F | 55 | - | + | - | + |

**Note:** M: male; F: female; +: positive; -: negative.


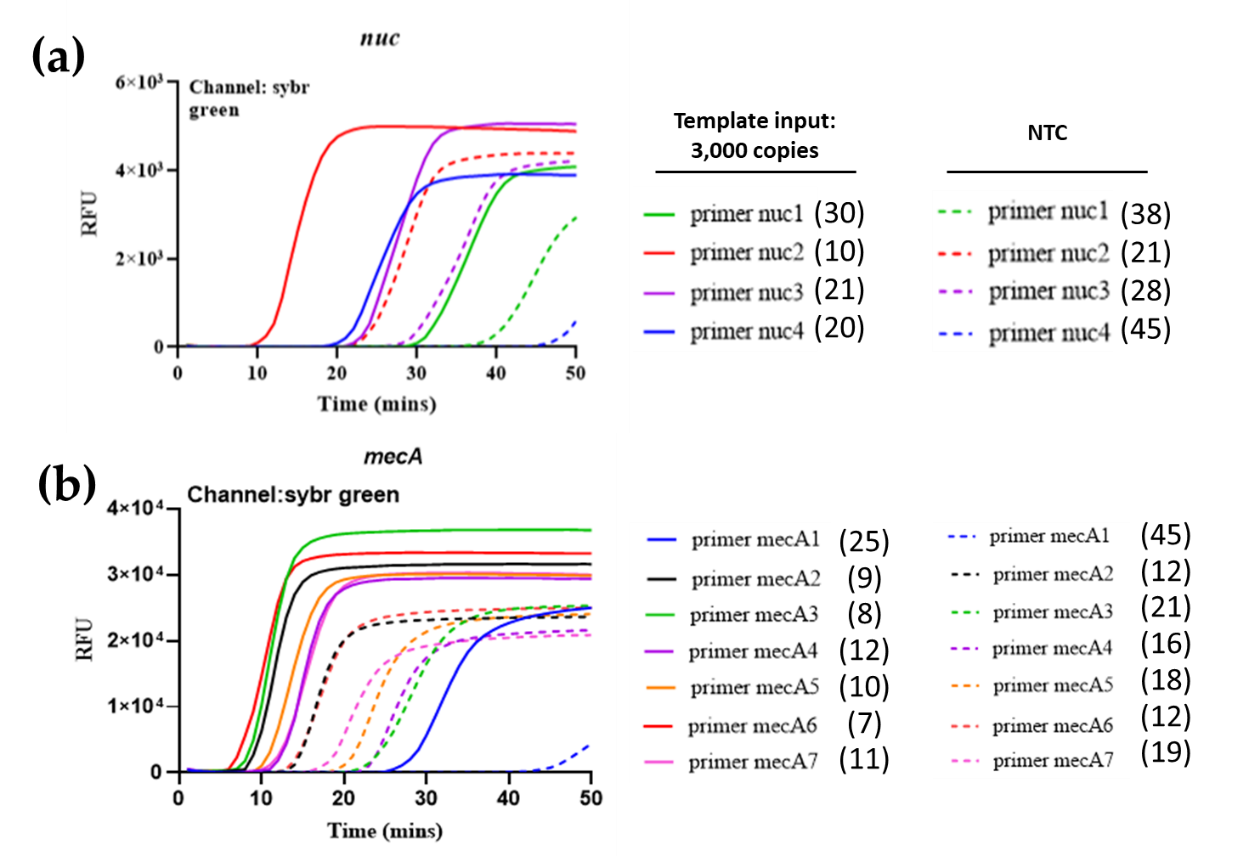


**Figure S1.** **Selection of the optimal LAMP primer sets for detection *nuc* (a) and *mecA* (b) genes.** NTC: non-template control. The Tt values are shown in parentheses.

| **(a)**  (*nuc* gene) |  |
| --- | --- |
| **(b)**  (mecA gene) | **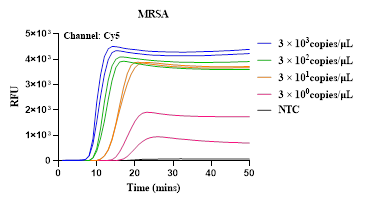** |

**Figure S2.** **Sensitivity of the single HiFi-LAMP assay for detection *nuc* (a) and *mecA* (b) genes.** NTC: non-template control.
